# Individualized Thalamic Parcellation Reveals Alterations in Shape and Microstructure of Thalamic Nuclei in Patients with Disorder of Consciousness

**DOI:** 10.1093/texcom/tgab024

**Published:** 2021-04-02

**Authors:** Weihao Zheng, Xufei Tan, Tingting Liu, Xiaoxia Li, Jian Gao, Lirong Hong, Xiaotong Zhang, Zhiyong Zhao, Yamei Yu, Yi Zhang, Benyan Luo, Dan Wu

**Affiliations:** Key Laboratory for Biomedical Engineering of Ministry of Education, College of Biomedical Engineering & Instrument Science, Zhejiang University, Hangzhou, 310027, P.R. China; Department of Clinical Medicine, School of Medicine, Zhejiang University City College, Hangzhou, 310015, P.R. China; Key Laboratory for Biomedical Engineering of Ministry of Education, College of Biomedical Engineering & Instrument Science, Zhejiang University, Hangzhou, 310027, P.R. China; Department of Neurology and Brain Medical Centre, The First Affiliated Hospital, School of Medicine, Zhejiang University, Hangzhou, 310003, P.R. China; Department of Rehabilitation, Hospital of Zhejiang Armed Police Corps, Hangzhou, 310051, P.R. China; Department of Rehabilitation, Hospital of Zhejiang Armed Police Corps, Hangzhou, 310051, P.R. China; Interdisciplinary Institute of Neuroscience and Technology, Qiushi Academy for Advanced Studies, Zhejiang University, Hangzhou, 310029, P.R. China; Center for Brain Imaging Science and Technology, College of Biomedical Engineering and Instrumental Science, Zhejiang University, Hangzhou, 310027, P.R. China; Key Laboratory for Biomedical Engineering of Ministry of Education, College of Biomedical Engineering & Instrument Science, Zhejiang University, Hangzhou, 310027, P.R. China; Department of Neurology and Brain Medical Centre, The First Affiliated Hospital, School of Medicine, Zhejiang University, Hangzhou, 310003, P.R. China; Key Laboratory for Biomedical Engineering of Ministry of Education, College of Biomedical Engineering & Instrument Science, Zhejiang University, Hangzhou, 310027, P.R. China; Department of Neurology and Brain Medical Centre, The First Affiliated Hospital, School of Medicine, Zhejiang University, Hangzhou, 310003, P.R. China; Key Laboratory for Biomedical Engineering of Ministry of Education, College of Biomedical Engineering & Instrument Science, Zhejiang University, Hangzhou, 310027, P.R. China

**Keywords:** disorder of consciousness, individualized thalamic parcellation, microstructural changes, nucleus deformation, thalamocortical connectivity

## Abstract

The thalamus plays crucial roles in consciousness generation and information processing. Previous evidence suggests that disorder of consciousness (DOC) caused by severe brain injury, is potentially related to thalamic abnormalities. However, how the morphology and microstructure change in thalamic subfields and thalamocortical fiber pathways in patients with DOC, and the relationships between these changes and the consciousness status remain unclear. Here, we generated the individual-specific thalamic parcellation in 10 DOC patients and 10 healthy controls (HC) via a novel thalamic segmentation framework based on the fiber orientation distribution (FOD) derived from 7-Tesla diffusion MRI, and investigated the shape deformation of thalamic nuclei as well as the microstructural changes associated with thalamic nuclei and thalamocortical pathways in patients with DOC. Enlargement of dorsal posterior nucleus and atrophy of anterior nucleus in the right thalamus were observed in DOC cohort relative to the HCs, and the former was closely linked to the consciousness level of the patients. We also found significant reductions of fiber density, but not fiber bundle cross-section, within several thalamic nuclei and most of the thalamocortical fiber pathways, suggesting that loss of axons might take primary responsibility for the impaired thalamocortical connections in patients with DOC rather than the change in fiber-bundle morphology. Furthermore, the individual-specific thalamic parcellation achieved 80% accuracy in classifying patients at the minimally conscious state from the vegetative state, compared with ~60% accuracy based on group-level parcellations. Our findings provide the first evidence for the shape deformation of thalamic nuclei in DOC patients and the microstructural basis of the disrupted thalamocortical connections.

## Introduction

Disorder of consciousness (DOC) is usually caused by severe brain injuries, such as hypoxic-ischemic encephalopathy and traumatic brain injury. Though patients may recover wakefulness (the presence of eye-opening and closing) after a period of coma, some of them only represent reflexive behavior without any behavioral signs of consciousness. Patients with such symptoms are defined as the vegetative state (VS; Jennett and Plum 1972). Some other patients may recover minor signs of consciousness, such as visual tracking, contextual emotional responses, and reproducible behaviors to commands. These patients are considered in a minimally conscious state (MCS; Giacino et al. 2002). Currently, over 40% of VS patients may be misdiagnosed by the neurobehavioral scale, such as the assessment of the coma recovery scale-revised (CRS-R; Schnakers et al. 2009), suggesting the difficulties of diagnoses determined by the clinical scale and the importance of finding objective and reliable neurological markers. Advances in magnetic resonance imaging (MRI) have allowed researchers to characterize structural and functional abnormalities associated with DOC, such as reduced gray matter (GM) and white matter (WM) integrities (Thibaut et al. 2015; Annen et al. 2018; Wang et al. 2018; Wu et al. 2018) and disrupted long-range functional and structural connections (Weng et al. 2017; Sinitsyn et al. 2018; Song et al. 2018b), which facilitate the diagnosis and prognostication for DOC (Rubeaux et al. 2015; Zheng et al. 2017; Song et al. 2018a; Wang et al. 2018). Nevertheless, the structural basis of loss and recovery of consciousness in severely brain-injured patients, a key question to the clinical management of DOC, remains largely unknown.

The thalamus is known as a key region that is extensively involved in the generation of consciousness (Alkire et al. 2008; Schiff 2008; Ward 2011; Liu et al. 2013; Mashour and Alkire 2013; Lutkenhoff et al. 2015). One plausible hypothesis of the role of the thalamus in consciousness generation is that the thalamus receives signals from primary sensory areas and projects them to high-order cortical associates for “computing” consciousness (Mumford 1991, 1992). The thalamic lesions are commonly observed in brain injury-induced loss of consciousness. Over 80% of the vegetative population may have thalamic damage (Adams et al. 2000), and the damages can be observed at both macro- and micro-level. For example, DOC was accompanied by volumetric atrophy (Fernández-Espejo et al. 2010; Rubeaux et al. 2015), reduced fractional anisotropy (FA), and increased diffusivity in the thalamus (Battistella et al. 2017; Najdenovska et al. 2018). Impaired cortico–thalamo–cortical circuitry was also reported in patients with DOC, especially between thalamus and posteromedial, sensorimotor, and frontal cortices (Fernández-Espejo et al. 2012; He et al. 2015; Weng et al. 2017), and these alterations were associated with the level of individual consciousness (Zheng et al. 2017, 2020). Besides, a previous study reported severer damage of the WM tracts connecting the subdivisions of the posteromedial cortex and thalamic nuclei in VS patients than in MCS patients (Cui et al. 2018). Together, previous studies pointed to alternations of the whole thalamus and disrupted thalamocortical connectivity in patients with DOC; however, how the structural changes of thalamic nuclei of individuals, in terms of their shape, connectivity pattern, and microstructural properties, are related to the DOC have not been well explored.

Existing evidence have suggested that patients with DOC were accompanied by volumetric reduction of the whole thalamus and deformation of its subfields (e.g., dorso-medial part) based on anatomical MRI (Fernández-Espejo et al. 2010), suggesting the atrophy of the thalamus may be related to its local changes. The “thalamic dynamic core” theory further posits that the consciousness may originate from the synchronous fluctuation of neurons in dorsal thalamic nuclei (Ward 2011). These studies pointed out the importance of understanding the relationship between alterations of thalamus subfields and the status of consciousness. We hypothesized that the DOC may be related to the structural alteration of specific thalamic nuclei that disturb the partitioning of the thalamus and alter the connectivity to the cortex, which, to our knowledge, has not been systematically examined. Previous studies regarding the subfields of thalamus mainly adopted a predefined thalamic atlas or the population atlas derived from a group of subjects, and have not investigated individual-specific shape deformation of thalamic nuclei for each subject. Since individualized segmentation would better characterize individual diversities in development, cognition, and behavior (Mueller et al. 2013; Han et al. 2019; Kong et al. 2019), it is desirable to generate individual-specific parcellation to study the abnormalities in thalamic subfields and the changes in pathways between thalamic nuclei and the cortex in DOC patients. A major challenge for analyzing the thalamic nuclei is that it requires reliable and robust thalamic parcels at the individual level. Several previous studies utilized features derived from diffusion MRI (dMRI), for example, diffusion tensor distance (Wiegell et al. 2003), the principal directions of the diffusion tensor (Ziyan et al. 2006; Mang et al. 2012; Kumar et al. 2015), and thalamocortical projections (Behrens et al. 2003; O'Muircheartaigh et al. 2011) for thalamic nuclei segmentation. The tensor-based features are known to have limited power in resolving crossing fibers (Jbabdi et al. 2010) and may not be sufficient in classifying the complex microstructures in the thalamus. Moreover, the low spatial resolution of dMRI, typically at in-plane resolution around 2 mm and slice thickness of 2–5 mm, has hampered the delineation of thalamic nuclei and their boundaries. Therefore, high-spatial and angular resolution dMRI is essential to improve the accuracy of segregating thalamic nuclei. The connectivity-based segmentation approaches, on the other hand, primarily focus on the major fiber pathways while ignoring the voxels containing small or crossing fibers; and also, a single voxel may send multiple pathways to different cortical areas which introduces ambiguity in segmentation. Recently, a novel approach for segmenting thalamic nuclei based on the spherical harmonic (SH) representation of fiber orientation distribution (FOD) was proposed (Battistella et al. 2017), which achieved high-intersubject consistency and high-intrasubject reproducibility in thalamic segmentation, and was robust to different sequences and scanners (Battistella et al. 2017; Najdenovska et al. 2018). Moreover, the spatial distribution of the segmented thalamic nuclei in vivo was in good accordance with Morel’s histological atlas (Morel et al. 1997). This approach laid the foundation for the investigation of individual-specific changes of thalamic nuclei in DOC, and the high-resolution dMRI on 7T may further enhance the advantage of the FOD-based segmentation approach.

Here, we analyzed multi-shell high-angular resolution dMRI (HARDI) data acquired on a 7-Tesla MRI scanner, to evaluate the DOC-related shape deformation of thalamic nuclei and the microstructural changes within thalamic nuclei and along the thalamocortical pathways in patients with DOC. Compared with the 3T MR, the 7T MR improves the spatial resolution and the signal-to-noise ratio (SNR; Vu et al. 2015), which is important for subnuclei segmentation and accurate and reliable reconstruction of the parameter maps (Luetzkendorf et al. 2012), for example, the habenula of thalamus could be better differentiated given the high contrast at 7T than 3T (Strotmann et al. 2014). With the advantage of high-spatial and angular resolutions, we computed the thalamic parcellation for each individual by using the FOD-based thalamic nuclei segmentation approach and estimated the shape distortion of each nucleus in DOC patients relative to the HCs. The individual-level thalamic parcellation was then utilized to characterize the individual macro- and micro-structural pathology of both the thalamic nuclei and thalamocortical pathways.

## Materials and Methods

### Participants

This study was approved by the local Research Ethics Committee of the First Affiliated Hospital of Zhejiang University School of Medicine, and written informed consents in accordance with the Declaration of Helsinki were obtained from healthy participants and the legal guardians of the patients to allow them to participate in the study and for this article to be published. Thirty-five patients with severe brain injuries were recruited from the Department of Rehabilitation in the Hangzhou Hospital of Zhejiang. CRS-R was utilized to evaluate the clinical condition of the participants on 3 consecutive days before 7 T MRI scan by 2 experienced clinicians (J.G. and L.H.). The inclusion criteria for DOC patients included: 1) disease course over 1 month but within 1 year; 2) no history of psychological disorders, epilepsy, or frequent spontaneous movements; 3) no alcohol, drug abuse, or use of the benzodiazepine class of drugs; 4) no MRI contraindications; and 5) no moderate or severe hydrocephalus or obvious brain lesion (examined by 1.5 T MRI scan). Eleven patients with MRI contraindications, 12 patients with extensive focal brain damage, and 1 patient diagnosed with the locked-in syndrome were excluded. Besides, 1 patient who awakened from a MCS before 7 T MRI scan was also kept out. Ten patients with DOC (10 males, 5 in VS/UWS, 5 in MCS; age range: 54.5 ± 11.7 years) and 10 healthy volunteers (9 males; age range: 43.1 ± 16.4 years) were finally included. There was no significant difference in age between the 2 groups (2-sample *t*-test, *P* > 0.05). Detailed clinical information of the patients was shown in Table 1.

**Table 1:** Detailed demographic and clinical characteristics of each patient

Patient ID	Age	Gender	Etiology	Scan time from onset (months)	CRS-R						CRS-R total
					A.	V.	M.	O.	C.	Ar.	
MCS1	56	M	TBI	7	2	3	4	0	0	2	11
MCS2	52	M	Anoxic	3.5	1	3	4	0	0	2	10
MCS3	50	M	HBI	5	2	3	3	2	1	3	14
MCS4	49	M	TBI	3	1	3	3	0	0	2	9
MCS5	68	M	HBI	8	1	3	1	1	0	2	8
VS1	72	M	TBI	3	0	0	1	1	0	2	4
VS2	68	M	TBI	3	1	1	2	1	0	2	7
VS3	52	M	TBI	1.5	0	0	2	0	0	2	4
VS4	42	M	HBI	5	0	2	1	0	0	2	5
VS5	36	M	HBI	4	0	0	2	0	0	2	4

*Abbreviations:* TBI: traumatic brain injury; HBI: hypoxic brain injury; A.: auditory; V.: visual; M.: motor; O.: oromotor; C.: communication; Ar.: arousal.

### MRI Acquisition

MRI data were acquired on a Siemens Magnetom 7 T scanner equipped with a Nova 1Tx/32Rx head coil. Multi-shell HARDI data were acquired with the following parameters: 1.25-mm isotropic voxels, acceleration factor = 2, echo time (TE) = 66.2 ms, repetition time (TR) = 5100 ms, flip angle = 90°, direction = 33 (for *b* = 1000 s/mm^2^), and 66 (for *b* = 2000 s/mm^2^). Both the nondiffusion-weighted and diffusion-weighted images were scanned with opposite phase-encoding directions (i.e., anterior-to-posterior and posterior-to-anterior) for the purpose of EPI distortion correction (Holland et al. 2010). T1-weighted images were obtained using magnetization-prepared rapid gradient echo (MPRAGE) sequence with 0.75-mm isotropic resolution, 208 slices, TE/TR = 2.51/2590 ms, inversion time = 1050 ms, and flip angle = 7°.

### Image Preprocessing

The preprocessing was conducted using MRtrix3 (www.mrtrix.org). All scans underwent denoising (Veraart et al. 2016), EPI distortion correction, corrections for eddy-current and motion (Andersson and Sotiropoulos 2016), bias field correction (Tustison et al. 2010), and normalization of intensities across subjects via the median intensities of WM, GM, and cerebrospinal fluid (CSF) of b0 images. Multi-shell multi-tissue (3 tissues) constrained spherical deconvolution algorithm was applied to compute the FODs based on the group averaged response functions for WM, GM, and CSF (Jeurissen et al. 2014). A population template was generated from FOD images of all subjects through iterative registration and averaging. The FOD images of all subjects were then registered to the population template using an FOD-guided nonlinear registration algorithm (Raffelt et al. 2011, 2012a). The exclusion of patients with obvious brain lesion that caused by TBI enhanced the registration performance from individual space to the population template (Weng et al. 2017). Notably, the nonlinear registration did not change the fiber orientations in the original FOD images. In addition, the population template for each of the 2 groups was also generated for comparison.

### Segmentation of Thalamic Nuclei for Individuals

**Figure 1:**
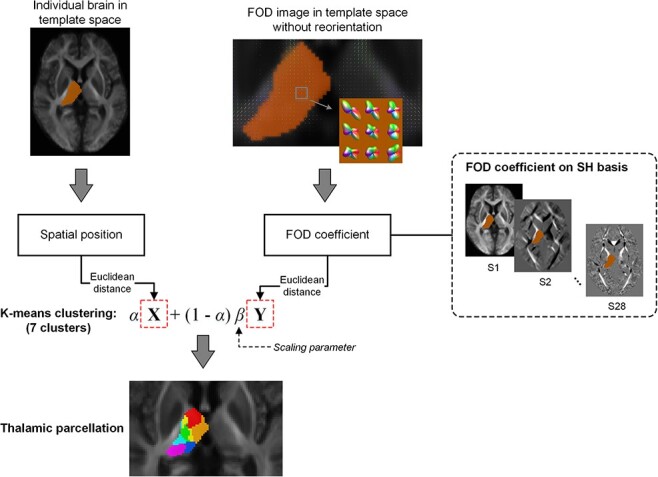
The pipeline of the thalamic nuclei segmentation. The Euclidean distance of spatial coordinates and the FOD coefficients (28 coefficients for each voxel) between pairs of voxels were calculated and combined with equal weights (*α* = 0.5). K-means clustering algorithm was used to segment each side of the thalamus to 7 nuclei.

We used an automatic thalamus segmentation algorithm with unsupervised k-means clustering based on FOD shape and spatial distance (Battistella et al. 2017; Najdenovska et al. 2018). The schematic overview of the segmentation algorithm is shown in Figure 1. The dMRI data were segmented using an online medical image preprocessing platform—MRIcloud (https://mricloud.org; Mori et al. 2016), by which the brains were segmented to 169 regions including the thalamus. The parcellation maps were warped to the population template using the transformation obtained from the FOD coregistration step (see Section Image Preprocessing). Then, the mask of the whole thalamus in the template space can be obtained. Both FOD coefficients and spatial distance between voxels within the thalamic mask were taken as inputs of the k-means clustering. In the present study, the maximum SH basis was set to 6, resulting in 28 FOD coefficients in each voxel. The metric for clustering is defined as:

$$ D=\alpha X+\left(1-\alpha \right)\beta Y $$

where *X* and *Y* are the Euclidean distance between FOD coefficients and between voxel coordinates, respectively; *α* determines the contribution of the 2 features, which was set to 0.5 as suggested by Battistella et al. (2017); and *β* is a scaling parameter that ensures *X* and *Y* are in the same scale, which was set at an empirical value (*β* = 85) based on the population template. We set the number of clusters to 7 for the thalamus on each hemisphere according to Behrens et al. (2003), O'Muircheartaigh et al. (2011), Battistella et al. (2017), and Najdenovska et al. (2018). The clustering centers were initialized using the centroids derived from hierarchical clustering based on only the spatial information.

### Parcellation Differences

Normalized mutual information (NMI) was used to quantify the similarity of thalamic partitions between the 2 groups. To make a group-level comparison, we calculated the consensus partition of the thalamus for each group using a consensus clustering algorithm (Lancichinetti and Fortunato 2012), which generates a stable group-level parcellation based on a set of individual partitions from the same group. The NMI is defined as:

$$ \mathrm{NMI}\left(A,B\right)=\frac{-2{\sum}_{i=1}^{M_A}{\sum}_{j=1}^{M_B}{N}_{ij}\log \left(\frac{N_{ij}N}{N_i{N}_j}\right)}{\sum_{i=1}^{M_A}{N}_i\log \left(\frac{N_i}{N}\right)+{\sum}_{j=1}^{M_B}{N}_j\log \left(\frac{N_j}{N}\right)} $$

where *A* and *B* are the parcellation of left/right thalamus of the 2 groups; *M_A_* and *M_B_* are the number of subregions in the parcellation; *N* is the number of voxels in the left/right thalamus; *N_i_* and *N_j_* represent the number of voxels in subregion *i* of *A* and subregion *j* of *B*, respectively; *N_ij_* is the number of voxels that module *i* and module *j* have in common. The NMI lies between 0 and 1, and a value close to 1 indicates a high similarity of parcellation between the 2 groups.

### Shape Analysis of Thalamic Nuclei

The shape information of thalamic nuclei was estimated by nonlinear registration of each thalamic subregion of individual DOC patient to the corresponding subregion in the population template of the HCs. The nonlinear registration was performed using FOD, which improves the alignment of WM bundles and thus provides more accurate registration (Raffelt et al. 2011, 2012a, 2017). The logarithm of the Jacobian determinant was utilized to represent the shape changes, with values > 0 indicating the expansion of subregions of DOC relative to the HC and values < 0 indicating opposite deformations. We further calculated the volumetric ratio changes of each thalamic nucleus in DOC patients relative to the HCs to examine whether the distortion of thalamic nuclei is related to the status of consciousness.

### Thalamo-Cortical Connectivity

To investigate changes in the thalamocortical pathway in patients with DOC and to identify the functions of the identified thalamic nuclei, a probabilistic fiber tracking algorithm (iFOD2) based on the second-order integration over FODs was applied to derive WM tracts between cortex and thalamus (Tournier et al. 2010). Five cortical seeds (i.e., prefrontal, motor, somatosensory, parietal-occipital, and temporal cortices) were created according to previous literature on the thalamocortical connection (Behrens et al. 2003; Nair et al. 2013). Fiber tracts originating from the cortical seeds and terminating in the thalamus were obtained with 2000 streamline samples for each cortical seed, tracing angle of 22.5°, the minimum/maximum fiber length of 10/250 mm, and the FOD cut-off value of 0.1. Because all the images have been coregistered to the population template, the same cortical seeds were used for individual fiber tracking. The fibers were then separated according to the connected thalamic nuclei that were identified based on the FOD of each subject. The track densities between the thalamic nuclei and cortical parcels were mapped back to the cortex for visualization of the contribution from the different nuclei. Notably, the last step was only performed on the population template by using the thalamic nuclei identified via the group averaging FOD.

### Fixel- and Voxel-Based Metrics

Both fixel- and voxel-based analyses were performed to investigate alterations in thalamic subfields as well as in thalamocortical connectivity in patients with DOC. For fixel-based analysis (Raffelt et al. 2017), we used 3 metrics including apparent fiber density (FD), fiber-bundle cross-section (FC), and the combination of FD and FC (FDC) (Raffelt et al. 2017). Briefly, FD is related to the restricted intra-axonal compartment, calculated as the integral of FOD along a certain orientation, and is proportional to the intra-axonal volume of fiber bundle aligned with that direction (Raffelt et al. 2012b). FC is a relative measure that estimates the individual differences in fiber bundle cross-section relative to the population template, which reflects the macrostructural (size) changes in fiber pathway (Raffelt et al. 2017). The FC perpendicular to the fixel direction is evaluated by nonlinear warping of an individual FOD image to the template image (Raffelt et al. 2017). FDC is computed as the product of FD and FC, representing combined effects to micro- and macro-structures. For voxel-based analysis, FA, mean diffusivity (MD), radial diffusivity (RD), and axial diffusivity (AD) within each voxel were extracted. All these metrics were averaged within each thalamic subfield or along each thalamocortical pathway.

### Comparison between Individual-Level and Population-Level Thalamic Parcellations

To investigate whether the parcellation at the single-subject level provides more specific information than using the group-level thalamic parcellation that was mapped onto the individual brains, we compared the classification performances of thalamic nuclei extracted from the individual- and population-level atlases to distinguish DOC patients and the subtypes. Two types of population atlases were utilized, including the thalamic partition obtained from the population FOD template and a predefined thalamic connectivity atlas (Behrens et al. 2003). The coregistration parameters between the thalamic connectivity atlas (in MNI space) and our population template were obtained by linear registration (affine) of the MNI152 T2 template to the population template through FMRIB’s linear image registration tool (FLIRT) (Jenkinson and Smith 2001; Jenkinson et al. 2002) followed by a nonlinear transformation through FMRIB’s nonlinear image registration tool (FNIRT) (Andersson et al. 2010). We performed 2 classification tasks (i.e., DOC vs. HC and MCS vs. VS) based on the combination of metrics (i.e., FD, FC, FDC, FA, MD, AD, and RD) of all the thalamic nuclei (but not thalamocortical fiber pathways), as well as the average metrics of the whole thalamus. The volume was excluded from the input features because the population-level parcellations led to the same nuclei volumes for all the individuals across groups. A sparse logistic regression model with L1-norm regularization was built as a classifier, and the classification performance was evaluated via a 3-fold cross-validation strategy that was repeated 20 times. In addition, the top 3 principal components extracted using principal component analysis of the features metrics were used for visualizing group differentiation.

### Statistical Analysis

Statistical tests were performed to evaluate the difference between DOC and HC groups in terms of the microstructural metrics of the thalamic nuclei and the corresponding fiber pathway, partition similarity, and predictive performances. To measure whether the shape changes of thalamic nuclei were statistically significant in patients with DOC relative to the HCs, one-sample *t*-test (with age, intracranial volume, and the duration of DOC status as covariates) was performed to test the average Jacobian determinant (log) of each nucleus. For microstructural metrics within nuclei and fiber pathways, we performed a nonparametric permutation test with 2000 permutations and controlled for the effects of age, intracranial volume, and the duration of DOC status. Multiple comparisons were corrected by false discovery rate (FDR) at the level of *q* = 0.05. For partition similarity, we randomly reallocated each subject to the 2 groups and recomputed the NMI between consensus partitions of the randomized groups. This procedure was repeated 1000 times to generate a distribution of NMI for the thalamus on each side, and 95% confidence interval of the distribution was used as critical values for a 2-tailed test at the threshold of *P* = 0.05. For the correlation with CRS-R scores, the partial correlation was utilized with age, intracranial volume, and the duration of DOC status as covariates.

## Results

### Segmented Thalamic Nuclei and their Connections to the Cortex

**Figure 2:**
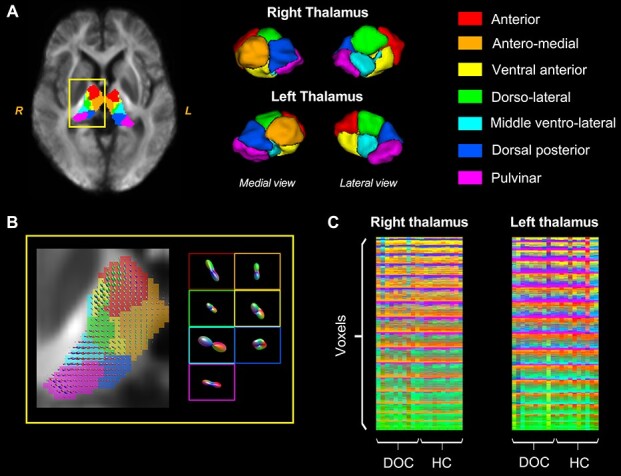
Visualization of the segmentation results. (*A*) Thalamic nuclei identified based on the FOD population template, as an example (segmentation on individual brains was not shown). (*B*) Thalamic FOD in the yellow rectangle in (*A*) and the average FOD of the 7 nuclei. (*C*) The concatenation of all thalamic voxels across individuals showed that the segmentation results were highly consistent across subjects. The color matrices illustrate the nuclei identity (1–7) of each voxel in the left or right thalamus for each of the subjects in the HC or DOC groups. Colors indicate different thalamic nuclei as shown in (*A*).

The segmentation result on the population template and the FOD information of each nucleus is visualized in Figure 2*A* and *B*, respectively. Global symmetric parcellation was observed between the bilateral thalamus. We identified the 7 thalamic nuclei based on their locations, including anterior (in red), antero-medial (in orange), ventral anterior (in yellow), dorso-lateral (in green), middle ventro-lateral (in light blue), dorsal posterior (in blue), and pulvinar (in fuchsia) parts. Although the parcellation exhibited individual differences, they maintained relatively high consistent topology across participants (Fig. 2*C*).

**Figure 3:**
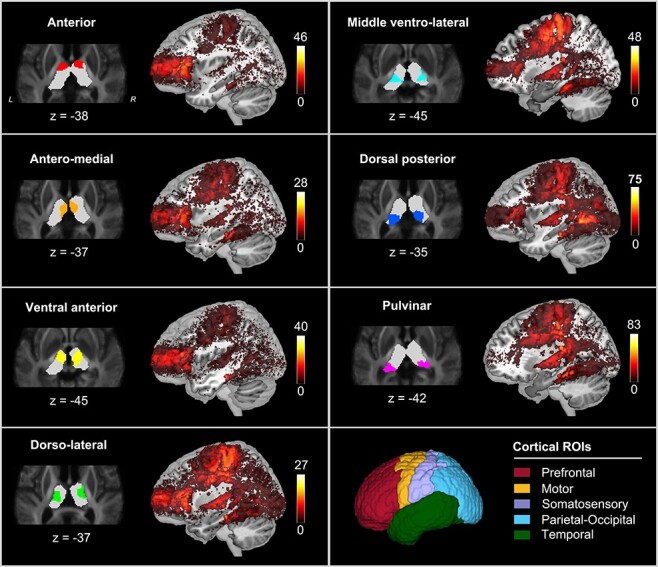
The track density images of fiber tracts that connected with each thalamic nucleus. Cortex is segmented into 5 regions of interest (ROI), including prefrontal, motor, somatosensory, parietal-occipital, and temporal cortices. The color map indicates the number of fibers connecting the cortical voxels.

The track density map indicates different connective patterns between each thalamic nucleus and the cerebral cortex (Fig. 3). Specifically, the 3 anterior nuclei (i.e., anterior, antero-medial, and ventral anterior) were mainly connected to the prefrontal cortex and a fraction of motor cortex; the dorso-lateral nuclei had a large number of connections with sensorimotor and prefrontal cortices; the middle ventro-lateral parts showed the strongest connectivity with motor cortex; the dorsal posterior nuclei primarily connected to the parietal-occipital cortex and also showed relatively high connectivity to motor cortex; the pulvinar nuclei were connected to both motor and temporal cortices. Our results were largely following previous connectivity-based parcellation defined by the “winner-take-all” principle that determined the thalamic nucleus by the target cortical area having the maximum proportion of connections with that nucleus (Behrens et al. 2003; Nair et al. 2013). For example, anterior, middle ventro-lateral, and dorsal posterior subfields of the thalamus can be roughly defined by their connection to prefrontal, motor, and parietal-occipital cortices, respectively.

### Alteration of the Thalamic Parcellation and Thalamic Nuclei Shapes in Individuals with DOC

**Figure 4:**
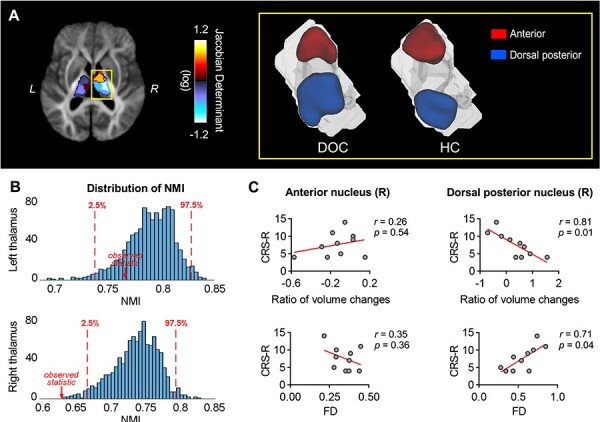
Comparison of the segmented thalamic nuclei between DOC and HC. (*A*) The extent of shape deformation in anterior (shrinkage) and dorsal posterior (expansion) nucleus of the right thalamus in patients with DOC relative to HC, as quantified by the logarithm of the Jacobian determinant. The yellow rectangle shows the template segmentation result of anterior and dorsal posterior nuclei of the right thalamus in the 2 groups. (*B*) Results from the permutation test of the NMI between thalamic partitions of DOC and HC groups. The histogram shows the distribution of NMI obtained from 1000 permutations. The similarity of right thalamic partitions between the 2 groups significantly decreased (*P* < 0.05). (*C*) Correlations between the CRS-R scores and the structural markers (FD and ratio changes of the volume relative to HC) in the 2 thalamic subfields with significant shape deformation. A significant negative correlation was found between the CRS-R scores and the volume changes in the right dorsal posterior nucleus (partial correlation with age, intracranial volume, and the duration of DOC status as covariates, *r* = 0.81, *P* = 0.01). In contrast, the FD showed a positive correlation with the CRS-R scores (partial correlation with age, intracranial volume, and the duration of DOC status as covariates, *r* = 0.71, *P* = 0.04). No significant correlation was found between the structural properties of the right anterior nucleus and the CRS-R scores (*Ps* > 0.05).

We further examined whether DOC significantly altered the parcellation architecture of the thalamus. By applying a consensus clustering algorithm, a group-level consensus partition for each cohort can be obtained (Supplementary Fig. S1). Parcellation differences between the 2 groups were found with a quantitative comparison. Particularly, significant deformation of right thalamus relative to the HCs, for example, enlargement of the dorsal posterior subfield and shrinkage of anterior subfield were identified (*P* < 0.05, FDR corrected, Fig. 4*A*). By performing permutation test on the NMI (see section Materials and Methods), significant dissimilarity between consensus partitions of the right thalamus (but not the left thalamus) was observed (*P* < 0.05, permutation test, Fig. 4*B*). These results indicated a greater morphological alteration of the right thalamus rather than the left side in patients with DOC.

We further examined the volumes and microstructural properties of these 2 nuclei and found the changes of volumetric ratio of the right dorsal posterior nucleus in the DOC cohort relative to the HCs showed a significant negative correlation with the CRS-R score (*r* = 0. 81, *P* = 0.01, Fig. 4*C*), which is consistent with our finding of expended right dorsal posterior nucleus in patients with DOC. We also found the average FD of the right dorsal posterior nucleus was positively correlated with the CRS-R score (*r* = 0.71, *P* = 0.04, Fig. 4*C*). Neither the volumetric nor the dMRI markers of the right anterior nucleus showed a significant correlation with the CRS-R scores (*Ps* > 0.05). These results implied a severe consciousness problem may be accompanied by the enlargement of the dorsal posterior area in the right thalamus rather than the left.

### Altered Microstructures in Patients with DOC

**Figure 5:**
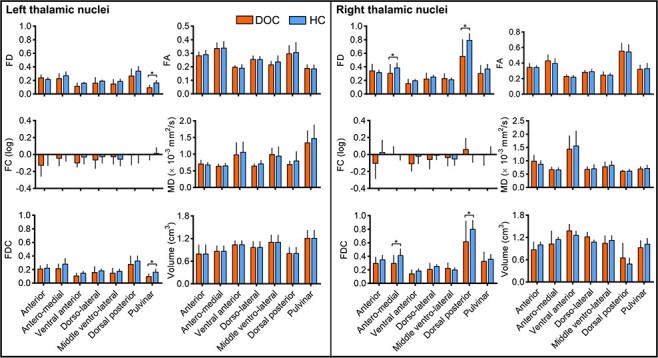
Changes of the macro- and micro-structural features of the thalamic nuclei in the DOC cohort, including FD, FC, FDC, FA, MD, and volumetric features. Significant reductions of the FD and FDC in the DOC group were found in right antero-medial and dorsal posterior parts, and left pulvinar regions (*qs* < 0.05, permutation test, FDR corrected). The changes of AD and RD were shown in Supplementary Fig. S2.

We tested for potential microstructural abnormalities in both thalamic subfields and thalamocortical fiber pathways in patients with DOC. For thalamic subfields, significant reductions of FD in patients with DOC were found in right antero-medial and dorsal posterior parts, and left pulvinar regions (*q* < 0.05, permutation test, FDR corrected, Fig. 5). Reduced FDC in these areas was also found in patients with DOC. However, no significant group difference was found in FC, FA, MD, AD, RD, and volume of thalamic subregions (*qs* > 0.05, FDR corrected, Fig. 5 and see Supplementary Fig. S2), indicating the density of axons may be more sensitive to the thalamic degeneration in DOC cohort, compared with conventional DTI-based markers.

**Figure 6:**
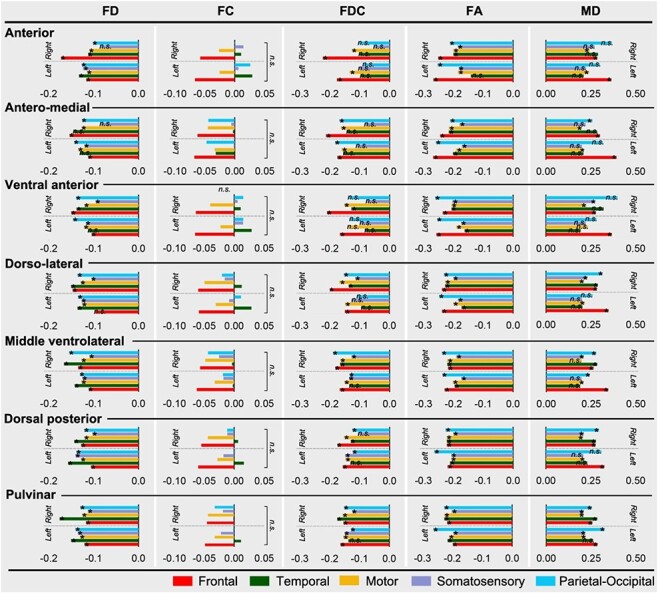
Percentage changes of the microstructural properties of the 5 thalamocortical pathways associated with each thalamic nuclei, in DOC patients relative to the HCs, including the FD, FC, FDC, FA, and MD measurements. Colors indicate the 5 cortical ROIs. The asterisk and *n.s.* indicates differences that survived FDR correction and nonsignificant results, respectively. The percentage changes of AD and RD were shown in Supplementary Fig. S3.

Significant reductions of FD, FDC, and FA in patients with DOC were also found along fiber pathways that connected thalamic nuclei to the 5 cortical areas (*q* < 0.05, permutation test, FDR corrected, Fig. 6). Such reductions were observed in almost all the pathways, except that the reductions of FDC in thalamocortical pathways that linked with temporal and somatosensory cortices were not statistically significant. We did not find a significant between-group difference in FC in any of the thalamocortical fiber pathways, but significant increases of MD, AD, and RD in the DOC group were observed (*q* < 0.05, permutation test, FDR corrected, Fig. 6 and see Supplementary Fig. S3), suggesting the increased diffusivity may be related to reduced axons within fiber bundles while the cross-section area of the bundles was relatively well-preserved.

### Diagnostic Performance Using Individualized Thalamic Parcellation Versus Population Atlas

**Figure 7:**
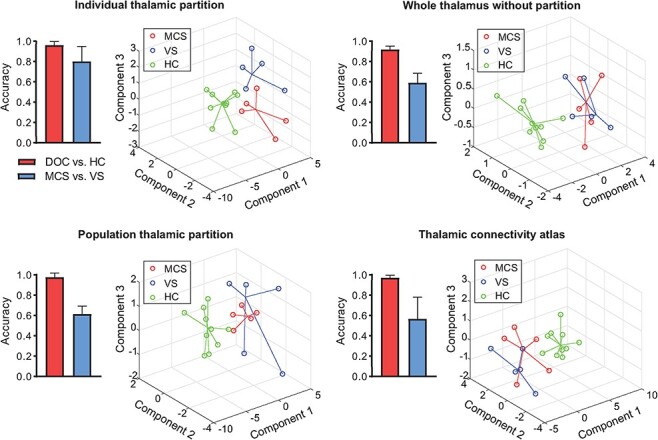
Comparisons of the classification performance using different thalamic partitions in identifying DOC from HC and MCS from VS. The classification accuracy of separating DOC and HC was improved by 6% when using the features of thalamic subfields, compared with the whole thalamus based classification. For the classification of MCS and VS, the accuracies were around 60% using the features of the whole thalamus or the features of thalamic subfields derived from a group-level parcellation (i.e., FOD template and thalamic connectivity atlas; Behrens et al. 2003). In contrast, the classification accuracy was improved to 80% when using the individualized thalamic partitions. The error bars represent the standard deviation of classification accuracies across the 3-fold cross-validation process of each classification task. The distribution of subjects represented in the feature space based on the top 3 principal components shows a clear boundary between DOC patients and controls and a larger separation between MCS and VS groups when using individualized thalamic partitions relative to the group-level partitions.

We investigated the advantage of individualized thalamic parcellation by comparing its classification performance with the whole-thalamus as well as population-level thalamic partitions based on the FOD template or the predefined thalamic connectivity atlas (Behrens et al. 2003). The results showed that classification using thalamic subfields (accuracies = 96.13%/97.62%/97.06% for individualized parcellation, the FOD template partition, and thalamic connectivity atlas, respectively) outperformed the whole thalamus (accuracy = 91.51%) in categorizing patients with DOC from the HCs (Fig. 7). However, the individual-level partition did not exhibit discriminative advances relative to population atlases in separating DOC and HC. For the task of distinguishing VS from MCS, the individual thalamic parcellation achieved the average accuracy of 80%, which outperformed both population-level parcellation (with accuracies of 61.39% and 56.67%, respectively, for the FOD template and connectivity-based population atlas) and the whole thalamus (accuracy of 58.89%), suggesting individual-level parcellation could better characterize the individual specific abnormalities and thus improved classification (Fig. 7). Also, features extracted from individual thalamic parcellation showed better separation between the MCS, VS, and HC populations than those from group-level atlases (Fig. 7).

## Discussion

How the thalamic structure and function modulate human consciousness has long been an open question. Previous studies focused on the role that the thalamus plays in modulating conscious awareness (Llinás 2003; Alkire et al. 2008; Ward 2011, 2013; Dehghani and Wimmer 2019). For example, a significant reduction in thalamic metabolism and blood flow during the anesthetics-induced loss of consciousness leads to the “thalamic switch” hypothesis, which assumes that the thalamus is considered as an on–off switch of consciousness (Alkire et al. 2000; Alkire and Miller 2005); another study showed that the spontaneous firing in the thalamus during anesthesia was driven by cortical neurons (Vahle-Hinz et al. 2007), indicating the thalamus may serve as a “readout” of cortical activity. Although the mechanism of how the thalamus modulates consciousness is not yet clear, the thalamus is undoubtedly one of the crucial substrates involved in the generation of consciousness. The present study aimed to examine the hypothesis that consciousness status of DOC patients is associated with the structural alteration of the thalamic nuclei. By using individual-specific thalamic partitions obtained from a novel data-driven thalamic segmentation approach based on 7 T high-field dMRI, both macro- and micro-level structural changes of the thalamic nuclei in patients with DOC were demonstrated. Substantial changes were observed in both macroscopic thalamic shapes and microscopic axonal properties, including 1) enlargement of the right dorsal posterior nucleus and shrinkage of the right anterior nucleus in individuals with DOC, 2) reduced axonal density in several thalamic nuclei and multiple thalamocortical pathways (accompanied by increased diffusivity), and 3) the changes of the nuclei could predict individual differences in state of consciousness. All these results supported our hypothesis, which, to our best knowledge, provides the first evidence for the altered macro- and micro-structural properties of thalamic nuclei in DOC cohort.

Most of the existing thalamic studies applied predefined atlases (e.g., the thalamic connectivity atlas [Behrens et al. 2003] and the thalamic probabilistic atlas [Iglesias et al. 2018]) to investigate the subregional structural changes, such as the GM density. However, the usage of predefined atlases leads to unified parcellation for all the subjects, which, therefore, overlooked the individual variability in thalamic partitions (e.g., the size and shape of the same thalamic nucleus may be different across individuals) and may not accurately reflect the influence of diseases on thalamic morphology. Recently, Han et al. found that the individual-specific parcels derived from WM tractography at the individual level generated more homogeneous connectivity pattern than the population atlas (Han et al. 2019), suggesting the advantage of the personalized parcellation in characterizing the individual differences. In the present study, we used a novel thalamic segmentation approach that generated a subject-specific partition based on the thalamic microstructure features (Battistella et al. 2017), which allows us to access potential alternation of the shapes of thalamic nuclei in DOC cohort. The multi-shell high field (7 T) dMRI with opposite phase encoding directions provided high-spatial resolution and signal-to-noise ratio, and improved the EPI distortion correction, which allowed accurate reconstruction of microstructural components that are important for the thalamic segmentation. The improved classification performance in distinguishing MCS and VS populations based on the individual thalamic parcellation supported the previous argument that individual-specific organization pattern captures the idiosyncrasies of subjects and better predicts human behavior (Kong et al. 2019; Li et al. 2019).

### Possible Reasons for the Altered Morphology of the Right Thalamic Nuclei in DOC Patients

The shape alterations of the anterior and dorsal posterior nuclei in the right thalamus in DOC patients were in good accordance with the deformation pattern reported in (Fernández-Espejo et al. 2010). These changes led to the decreased similarity of segmented parcels of the right thalamus between patients with DOC and the HCs. Because the segmentation algorithm was based on the FOD within each voxel, the deformation of these 2 subfields may result from increased/decreased number of voxels with similar fiber orientations. Since the anterior and dorsal posterior subfields were mainly connected with transmodal (e.g., frontal cortex) and unimodal cortices (e.g., sensory, auditory, and visual cortices), respectively, these changes reflected the reduced number of voxels that connected to the frontal cortex and increased connections to the perception cortices, which may indirectly reflect alterations of cortico–thalamo–cortical loops in patients with DOC that were pivotal for the integration of bottom-up (e.g., driven by sensory inputs) and top-down (e.g., driven by context and computations from frontal and association cortices) processes (Mumford 1991, 1992; Zikopoulos and Barbas 2006; Jones 2009; Parnaudeau et al. 2018). These abnormalities, we speculated, may result from the compensatory mechanisms of the WM (Kuchtova et al. 2018; Sanjari Moghaddam et al. 2020). The expansion of the right posterior thalamus may reflect the compensation of pathways to unimodal primary areas by scarifying the connections to transmodal areas. Since the “computation” of consciousness in the high-order cortical regions (Mumford 1991, 1992; Velly et al. 2007) is determined by the sensory inputs (Silvanto et al. 2005), the thalamocortical connections may be prioritized to relay the sensory signals rather than the association signals.

Other interpretations for the alteration of FOD were also possible. For example, the WM structure may be dynamically modulated by the external condition or self-demand (Schlaug et al. 2009; Sampaio-Baptista and Johansen-Berg 2017), and by axonal activity (Stevens et al. 1998; Ishibashi et al. 2006; Hines et al. 2015) that were reduced in both thalamic and cortical regions in DOC patients (Hannawi et al. 2015; Magrassi et al. 2018). However, since there were very few studies that directly investigated the volumetric change of thalamic nuclei in patients with DOC, its biological substrate needs to be explored in future studies.

Interestingly, significant subfield deformation, accompanied by the reorganized parcellation, was only observed in the right thalamus, suggesting the hemispheric asymmetry of thalamic damage in the DOC cohort. Previous studies have showed higher concentration of norepinephrine in the right thalamus than the left (Oke et al. 1978, 1980), indicating the norepinephrine-dependent activity—arousal and maintenance of attention by sensory perception (Tucker and Williamson 1984) that is indispensable for shaping consciousness (Chakrabarty et al. 2017) was more active in the right hemisphere. Besides, the right dominance of prefrontal activation in poststroke patients with DOC (Moriya and Sakatani 2018) and the rightward lateralization of excitatory interactions during consciousness organization (Velichkovsky et al. 2018) suggested the preferential contribution of the right hemisphere towards consciousness, which was hampered in DOC patients. These studies supported our findings of the right-lateralized thalamic alterations in patients with DOC.

### Altered Macroscopic and Microscopic Structures of the Right Dorsal Thalamic Nuclei Predicts the Status of Consciousness in DOC Patients

In the 2 subregions with significant morphological changes, only the structural properties of the right dorsal posterior nucleus (i.e., volumetric and FD changes) were able to predict the status of consciousness in the DOC cohort; whereas no significant correlation was found between structural changes of the right anterior nuclei and the CRS-R scores, providing evidence for the dorsal thalamic area might play a key role in consciousness generation. This was in line with previous literature indicating the consciousness may be generated from synchronized neural activity of dorsal thalamic nuclei (Ward 2011), and the recovery of consciousness was not related to the restoration of thalamo-frontal connectivity (Crone et al. 2018). However, opposite alteration trends were found between the macro- and micro-structures of the right dorsal posterior nucleus in the DOC cohort, for example, severer consciousness impairment was accompanied by its volume enlargement but FD reduction. These were not necessarily conflicted results because the 2 metrics reflect different information (Raffelt et al. 2017). Specifically, FD measures the number/density of the axons within a fiber bundle (Raffelt et al. 2012b), whereas volume is a more macroscopic measure reflecting the number of voxels in the nucleus. The compensatory mechanism may also apply to the relation between the shape and FD changes, for example, the decreased FD in fiber bundles related to the dorsal posterior subfield may be compensated by an increased number of voxels with similar fiber orientations to maximally restore the communication with sensory, auditory, and visual cortices, resulting in the volumetric expansion of these nuclei. This viewpoint was supported by a previous study indicating loss of projection from the hippocampal formation in patients with Alzheimer’s disease was compensated by hypertrophy of related fibers (Kuchtova et al. 2018). The positive correlation between FD and the CRS-R score suggested DOC was associated with loss of axons in the dorsal area of the right thalamus, consistent with a previous study indicating the reduction of neurons in dorso-medial thalamic nucleus led to lower outcomes of consciousness assessment in moderately and severely disabled and vegetative patients (Maxwell et al. 2004).

### Abnormal Thalamocortical Connection in DOC Patients Is Associated with Reduced Axonal Density

We found significant reductions of FD in most of the thalamocortical fiber pathways in patients with DOC, though some of the related thalamic nuclei did not show evident microstructural changes. Accumulating evidence has suggested that loss of consciousness is associated with disconnections in the brain (Boveroux et al. 2010; Boly et al. 2012; Uehara et al. 2014; Song et al. 2018b; Ferraro et al. 2019). Especially, long-range connections, for example, thalamocortical connections, are vulnerable to brain injury (Alkire et al. 2008; Yao et al. 2015; Weng et al. 2017; Zheng et al. 2017; Crone et al. 2018). Although previous studies have shown decreased tract integrity (characterized by FA) between the posteromedial cortex and the thalamus in MCS and VS/UWS patients (Fernández-Espejo et al. 2012; Cui et al. 2018), our study moves a further step to elucidate that the disrupted thalamocortical connectivity in DOC may be primarily associated with extensive loss of axons within fiber bundles (characterized by reduced FD) rather than the atrophy of fiber tracts (characterized by the nonsignificant changes in FC). Such alteration may impede the signal conduction efficiency within cortico–thalamo–cortical loops, resulting in loss of consciousness. In addition, some previous studies implied that the increased WM diffusivity in DOC patients may result from demyelination (Wang et al. 2018; Wu et al. 2018), our results provided another direct interpretation that the increased diffusivity was related to FD reductions that result in more free water diffusion in fiber bundles.

### Limitation

A major limitation lies in the limited sample size in the present study (*n* = 10). Although some of our findings were in line with previous literature, the small sample size reduced the reliability of the results. The findings needed to be examined on a large cohort in future work. In addition, we did not separate DOC patients by their pathogeneses (i.e., hypoxic–ischemic encephalopathy and traumatic brain injury) because of the sample size. It is known that these 2 causes may have different representations of WM injury (van der Eerden et al. 2014). Further study needs to be performed on a larger cohort and separate HIE and TBI patients to examine the macro- and micro-level thalamic structural changes between groups. Moreover, we utilized the same parameters for thalamus segmentation for all the participants. Though segmentation results were in good accordance across participants, the parameters may not be optimal for all the participants, and an optimization method needs to be developed to find the optimal parameters for individual thalamic segmentation. This is beyond the scope of the present study, but could be investigated in future work via the existing optimization algorithms (e.g., the genetic algorithm [Holland 1984]). In addition, though we excluded the patients with obvious brain lesion to reduce the influence of lesion site on image analysis, the relationship between lesion site and the damage pattern of the thalamus of patients with DOC is still an interesting topic and will be investigated in the future work.

## Conclusion

In this study, we used a novel FOD-based thalamic nuclei segmentation approach to obtain the individual-specific thalamic parcellation based on 7 T high-resolution diffusion data, which showed unique advantages in distinguishing the consciousness status of DOC patients. We found that individuals with DOC showed remarkable morphological changes in the anterior and dorsal posterior parts of the right thalamus, especially, the volume of the dorsal posterior nucleus was associated with the status of consciousness of the patients. Furthermore, significant reductions of FD (but not FC) within most of the fiber pathways between the thalamic nuclei and cerebral cortex indicate that loss of axons may be the primary reason for the disruption of thalamocortical connectivity in DOC patients rather than the alteration of fiber-bundle morphology. Our findings reveal the individual difference of thalamic structural changes in patients with DOC, from macroscopic to microscopic level, and provide direct microstructural interpretation for the abnormal thalamocortical connectivity in the DOC cohort.

## Supplementary Material

supplementary_tgab024

## Data Availability

The datasets generated during and/or analyzed during the current study are not publicly available, but are available from the corresponding author on reasonable request.
