# Supplementary material for: Individualized Thalamic Parcellation Reveals Alterations in Shape and Microstructure of Thalamic Nuclei in Patients with Disorder of Consciousness
Source: Cereb Cortex Commun. 2021 Apr 2;2(2):tgab024. doi: 10.1093/texcom/tgab024 (PMC8152869; doi:10.1093/texcom/tgab024)
Supplement: supplementary_tgab024 [file supplementary_tgab024.docx]

**Supplementary**


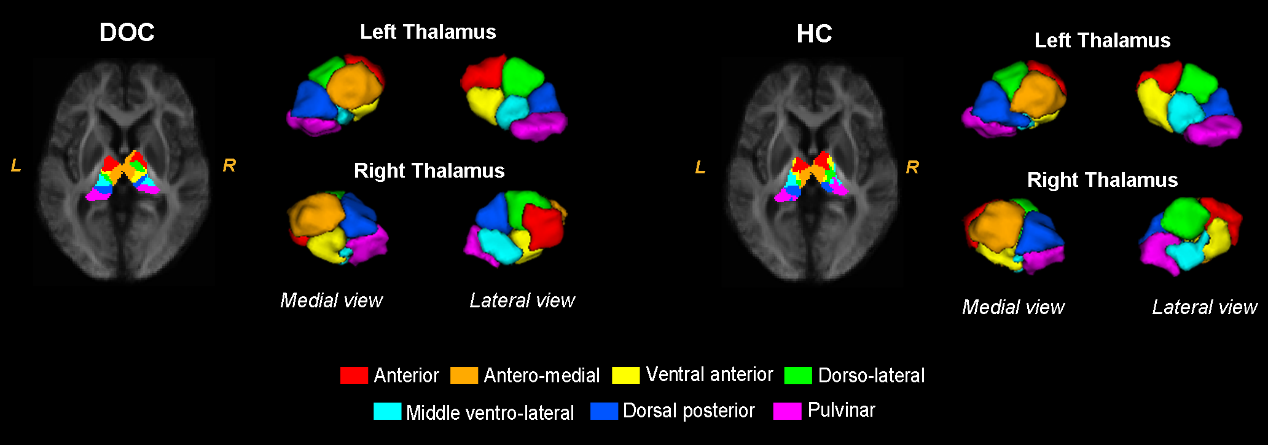


**Figure S1.** The consensus partitions of the thalamus for DOC and HC group, respectively. Colors indicate different thalamic nuclei.


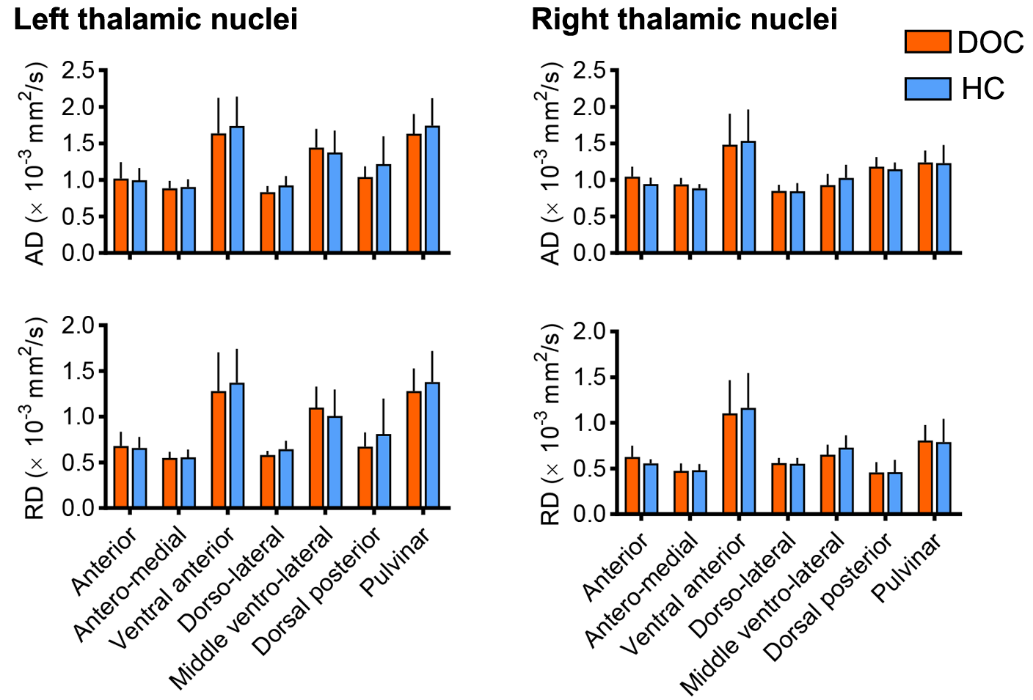


**Figure S2.** Changes of axial diffusivity (AD) radial diffusivity (RD) of the thalamic nuclei in the DOC cohort.


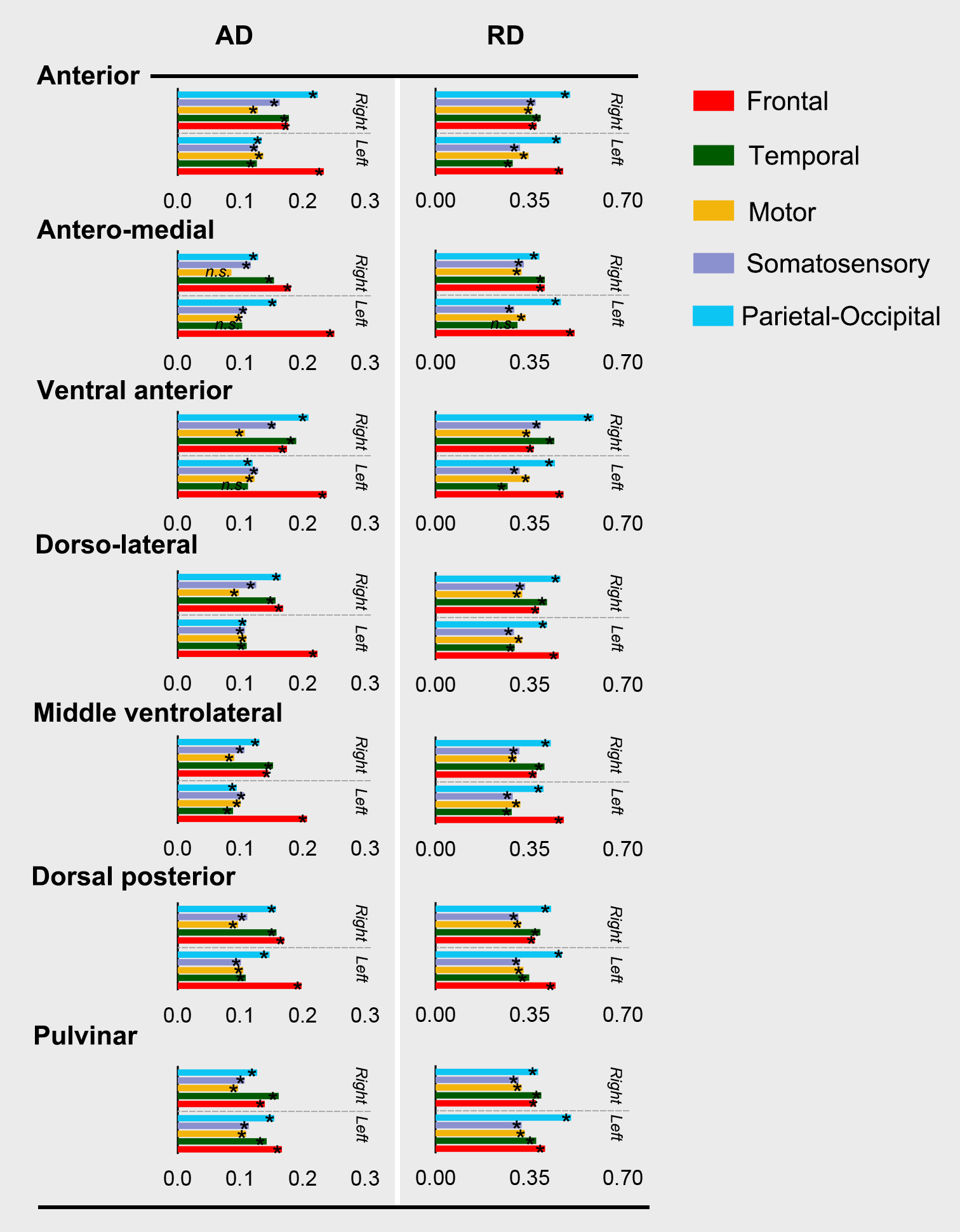


**Figure S3.** Percentage changes of axial diffusivity (AD) radial diffusivity (RD) of the five thalamocortical pathways associated with each thalamic nuclei, in DOC patients relative to the HCs. Colors indicate the five cortical ROIs. The asterisk and *n.s.* indicates differences that survived from FDR correction and non-significant results, respectively.


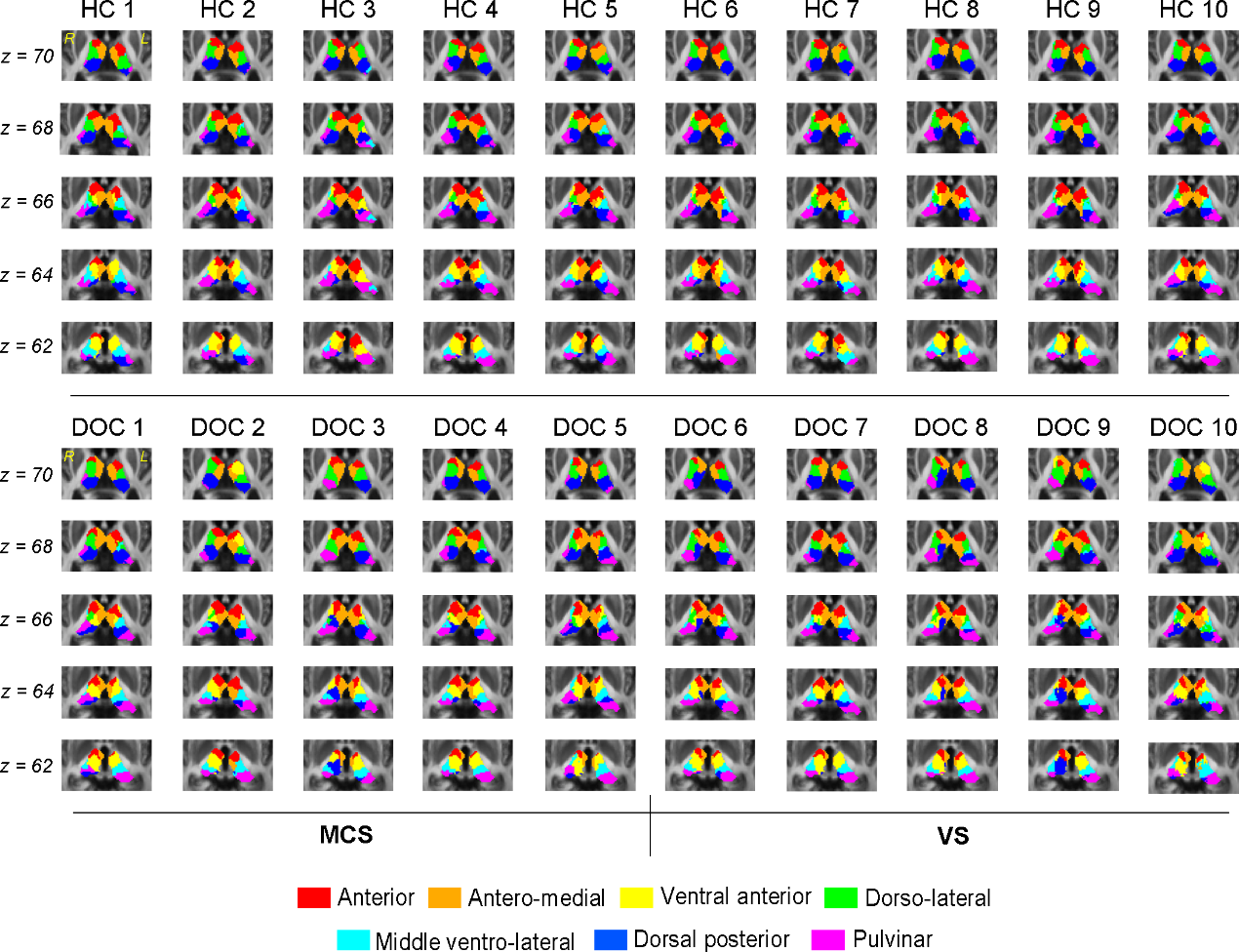


**Figure S4.** Visualization of the thalamic parcellation of each individual. Colors indicate different thalamic nuclei.
